# Supplementary material for: Modelling Skylarks (Alauda arvensis) to Predict Impacts of Changes in Land Management and Policy: Development and Testing of an Agent-Based Model
Source: PLoS One. 2013 Jun 6;8(6):e65803. doi: 10.1371/journal.pone.0065803 (PMC3675089; doi:10.1371/journal.pone.0065803)
Supplement: Supporting Information S4 — The skylark ODdox as a zipped archive. (ZIP) [file pone.0065803.s004.zip › Skylark_ODdox/tole__declaration_8h.html]

ALMaSS Skylark ODdox: tole\_declaration.h File Reference


ALMaSS Skylark ODdox
 2.0


- Main Page
- Related Pages
- Classes
- Files

- File List
- File Members

Enumerations

tole\_declaration.h File Reference

|  |  |
| --- | --- |
| Enumerations | |
| enum | TTypesOfLandscapeElement {     tole\_Hedges = 0, tole\_RoadsideVerge, tole\_Railway, tole\_FieldBoundary,     tole\_Marsh, tole\_Scrub, tole\_Field, tole\_PermPastureLowYield,     tole\_PermPastureTussocky, tole\_PermanentSetaside, tole\_PermPasture, tole\_NaturalGrass,     tole\_RiversidePlants, tole\_PitDisused, tole\_RiversideTrees, tole\_DeciduousForest,     tole\_MixedForest, tole\_ConiferousForest, tole\_YoungForest, tole\_StoneWall,     tole\_Garden, tole\_Track, tole\_SmallRoad, tole\_LargeRoad,     tole\_Building, tole\_Urban, tole\_ActivePit, tole\_Freshwater,     tole\_River, tole\_Saltwater, tole\_Coast, tole\_HedgeBank,     tole\_BeetleBank, tole\_Heath, tole\_Orchard, tole\_UnsprayedFieldMargin,     tole\_OrchardBand, tole\_OrchardGrass, tole\_BareRock, tole\_AmenityGrass,     tole\_Parkland, tole\_UrbanNoVeg, tole\_UrbanPark, tole\_Suburban,     tole\_RuralResidential, tole\_BuiltUpWithParkland, tole\_SandDune, tole\_Copse,     tole\_Foobar   } |

---

## Enumeration Type Documentation

enum TTypesOfLandscapeElement

**Enumerator:**
:   |  |  |
    | --- | --- |
    | *tole\_Hedges* |  |
    | *tole\_RoadsideVerge* |  |
    | *tole\_Railway* |  |
    | *tole\_FieldBoundary* |  |
    | *tole\_Marsh* |  |
    | *tole\_Scrub* |  |
    | *tole\_Field* |  |
    | *tole\_PermPastureLowYield* |  |
    | *tole\_PermPastureTussocky* |  |
    | *tole\_PermanentSetaside* |  |
    | *tole\_PermPasture* |  |
    | *tole\_NaturalGrass* |  |
    | *tole\_RiversidePlants* |  |
    | *tole\_PitDisused* |  |
    | *tole\_RiversideTrees* |  |
    | *tole\_DeciduousForest* |  |
    | *tole\_MixedForest* |  |
    | *tole\_ConiferousForest* |  |
    | *tole\_YoungForest* |  |
    | *tole\_StoneWall* |  |
    | *tole\_Garden* |  |
    | *tole\_Track* |  |
    | *tole\_SmallRoad* |  |
    | *tole\_LargeRoad* |  |
    | *tole\_Building* |  |
    | *tole\_Urban* |  |
    | *tole\_ActivePit* |  |
    | *tole\_Freshwater* |  |
    | *tole\_River* |  |
    | *tole\_Saltwater* |  |
    | *tole\_Coast* |  |
    | *tole\_HedgeBank* |  |
    | *tole\_BeetleBank* |  |
    | *tole\_Heath* |  |
    | *tole\_Orchard* |  |
    | *tole\_UnsprayedFieldMargin* |  |
    | *tole\_OrchardBand* |  |
    | *tole\_OrchardGrass* |  |
    | *tole\_BareRock* |  |
    | *tole\_AmenityGrass* |  |
    | *tole\_Parkland* |  |
    | *tole\_UrbanNoVeg* |  |
    | *tole\_UrbanPark* |  |
    | *tole\_Suburban* |  |
    | *tole\_RuralResidential* |  |
    | *tole\_BuiltUpWithParkland* |  |
    | *tole\_SandDune* |  |
    | *tole\_Copse* |  |
    | *tole\_Foobar* |  |

{

tole\_Hedges=0, // 130

tole\_RoadsideVerge, // 13

tole\_Railway, // 118

tole\_FieldBoundary, // 160

tole\_Marsh, // 95

tole\_Scrub, // 70

tole\_Field, // 20 & 30

tole\_PermPastureLowYield, // 26

tole\_PermPastureTussocky, // 27

tole\_PermanentSetaside, // 33

tole\_PermPasture, // 35

tole\_NaturalGrass, // 110 //tole 11

tole\_RiversidePlants, // 98

tole\_PitDisused, // 75

tole\_RiversideTrees, // 97

tole\_DeciduousForest, // 40

tole\_MixedForest, // 60

tole\_ConiferousForest, // 50

tole\_YoungForest, //55

tole\_StoneWall, // 15

tole\_Garden, //11 // tole 20

tole\_Track, // 123

tole\_SmallRoad, // 122

tole\_LargeRoad, // 121

tole\_Building, // 5

tole\_Urban, // 10

tole\_ActivePit, // 115

tole\_Freshwater, // 90

tole\_River, // 96

tole\_Saltwater, // 80

tole\_Coast, // 100 // tole 30

tole\_HedgeBank, // 140

tole\_BeetleBank, // 141

tole\_Heath, // 94

tole\_Orchard, //56

tole\_UnsprayedFieldMargin, // 31

tole\_OrchardBand, // 57

tole\_OrchardGrass, //58

tole\_BareRock, // 59

tole\_AmenityGrass, // 12

tole\_Parkland, // 14

tole\_UrbanNoVeg, // 6

tole\_UrbanPark, // // 17

tole\_Suburban, // 7

tole\_RuralResidential, // 8

tole\_BuiltUpWithParkland, // 16

tole\_SandDune, //101

tole\_Copse, // 41

tole\_Foobar // 999 !! type unknown - should not happen

// Don't use this one when communicating with the landscape

// model, it will trip an error in most cases!

// --FN-- 24/11-2000.

}


- CJT
- MSVC
- ALMaSS Working Source
- Landscape
- tole\_declaration.h
- Generated on Thu Jan 10 2013 13:15:35 for ALMaSS Skylark ODdox by
   1.8.1.1
